# Concurrent Training Promotes Greater Gains on Body Composition and Components of Physical Fitness Than Single-Mode Training (Endurance or Resistance) in Youth With Obesity

**DOI:** 10.3389/fphys.2022.869063

**Published:** 2022-05-20

**Authors:** Marwa Bouamra, Hassane Zouhal, Sébastien Ratel, Issam Makhlouf, Ikram Bezrati, Mokhtar Chtara, David G. Behm, Urs Granacher, Anis Chaouachi

**Affiliations:** ^1^ Tunisian Research Laboratory “Sports Performance Optimization”, National Center of Medicine and Science in Sports (CNMSS), Tunis, Tunisia; ^2^ High Institute of Sport and Physical Education, Ksar-Saïd, Manouba University, Tunis, Tunisia; ^3^ M2S (Laboratoire Mouvement, Sport, Santé) EA 1274, University of Rennes, Rennes, France; ^4^ Institut International des Sciences du Sport (2I2S), Irodouer, France; ^5^ AME2P, EA 3533, Clermont-Auvergne University, Clermont-Ferrand, France; ^6^ School of Human Kinetics and Recreation, Memorial University of Newfoundland, St. John’s, NL, Canada; ^7^ Division of Training and Movement Sciences, University of Potsdam, Potsdam, Germany; ^8^ High Institute of Sport and Physical Education, Sfax University, Sfax, Tunisia; ^9^ Sports Performance Research Institute New Zealand, AUT University, Auckland, New Zealand

**Keywords:** weight loss, adolescents, high-intensity-interval training, resistance training, DXA, matched time

## Abstract

The prevalence of obesity in the pediatric population has become a major public health issue. Indeed, the dramatic increase of this epidemic causes multiple and harmful consequences, Physical activity, particularly physical exercise, remains to be the cornerstone of interventions against childhood obesity. Given the conflicting findings with reference to the relevant literature addressing the effects of exercise on adiposity and physical fitness outcomes in obese children and adolescents, the effect of duration-matched concurrent training (CT) [50% resistance (RT) and 50% high-intensity-interval-training (HIIT)] on body composition and physical fitness in obese youth remains to be elucidated. Thus, the purpose of this study was to examine the effects of 9-weeks of CT compared to RT or HIIT alone, on body composition and selected physical fitness components in healthy sedentary obese youth. Out of 73 participants, only 37; [14 males and 23 females; age 13.4 ± 0.9 years; body-mass-index (BMI): 31.2 ± 4.8 kg·m-2] were eligible and randomized into three groups: HIIT (*n* = 12): 3-4 sets×12 runs at 80–110% peak velocity, with 10-s passive recovery between bouts; RT (*n* = 12): 6 exercises; 3–4 sets × 10 repetition maximum (RM) and CT (*n* = 13): 50% serial completion of RT and HIIT. CT promoted significant greater gains compared to HIIT and RT on body composition (*p* < 0.01, d = large), 6-min-walking test distance (6 MWT-distance) and on 6 MWT-VO_2max_ (*p* < 0.03, d = large). In addition, CT showed substantially greater improvements than HIIT in the medicine ball throw test (20.2 *vs*. 13.6%, *p* < 0.04, d = large). On the other hand, RT exhibited significantly greater gains in relative hand grip strength (*p* < 0.03, d = large) and CMJ (*p* < 0.01, d = large) than HIIT and CT. CT promoted greater benefits for fat, body mass loss and cardiorespiratory fitness than HIIT or RT modalities. This study provides important information for practitioners and therapists on the application of effective exercise regimes with obese youth to induce significant and beneficial body composition changes. The applied CT program and the respective programming parameters in terms of exercise intensity and volume can be used by practitioners as an effective exercise treatment to fight the pandemic overweight and obesity in youth.

## 1 Introduction

The World Health Organization (WHO) recommends youth aged 5–17 years to be physically active on average at least 60 min per day at moderate-to-vigorous intensities to improve muscular fitness, bone health, and cardiovascular and metabolic health biomarkers (Bull et al., 2020). More recent physical activity (PA) guidelines have recommended a minimum dosage of 90 min per day, which should include daily aerobic activities (Jamka et al., 2021) and muscle strengthening exercises at least 3 times per week. However, only 20% of youth follow the WHO PA guidelines (Hardy et al., 2017). Low PA levels with excessive food intake result in an energy imbalance that could lead to overweight or obesity (Hsu et al., 2021). Data from 12 European countries indicate prevalence rates of youth overweight (+1 SD from mean body mass index (BMI) z score) and obesity (+2 SD from mean BMI z score) ranging from 19.3 to 49.0% for overweight boys and 18.4–42.5% for girls as well as from 6.0 to 26.6% for obese boys and 4.6–17.3% for girls respectively (Hruby and Hu, 2015). Tunisia is a middle-income developing North African country with high prevalence rates for overweight and obesity in youth. Between 1996 and 2005, 1.5 to 5.0-fold increases in prevalence rates for overweight were noted for girls and boys respectively (for a review see Ammar et al. (2015). Currently, overweight and obesity rates range between 11.6 to 48.9% and 2.7–10.0%, respectively depending on age, sex, and region within Tunisia. The increase in childhood and adolescent overweight and obesity have been attributed to increased sedentarism, physical inactivity factors, which are moderated by the individual’s socio-economic family background (Maatoug et al., 2013) causing long-term imbalances between energy intake and energy expenditure (Maatoug et al., 2013). For Tunisia, there is evidence that the risk of suffering from overweight or obesity is higher for youth from families of high socio-economic status. There is evidence that school-aged children in Tunisia suffer from poor diet, physical inactivity, and increased screen time (Maatoug et al., 2013; Cherif et al., 2018). For example, Gaha et al. (2002) reported that the prevalence for overweight and obesity is particularly high in physically inactive children. The same authors reported that excessive weight is more prevalent (9%) in children who do not practice sport at school and are a member of a sport club compared with active children who adhere to WHO recommendations of at least 60 min daily physical activity. Of note, a meta-analysis revealed that physical inactivity, increased screen time, and higher socio-economic status were risk factors for childhood obesity in the Middle East and North Africa (MENA) (Farrag et al., 2017). Therefore**,** obesity is characterized as a global epidemic that affects all ages (Ghouili et al., 2018) with childhood and adolescence being crucial periods for prevention and intervention efforts (Monteiro et al., 2015). Moreover, it is often coupled with impairments in cardiovascular fitness, muscle strength, physical function, and the capacity to perform daily activities (Pazzianotto-Forti et al., 2020). So, PA, especially exercise training, remains to be a cornerstone of pediatric obesity management (Schranz et al., 2013; Zouhal et al., 2020). Some of the primary goals of exercise in the treatment and prevention of obesity are to promote body fat loss and improvements in functional capacity and cardiorespiratory fitness. Better cardiorespiratory capacity enhances cardiometabolic markers, optimizes cardiovascular health, and decreases the risk of mortality in the long term (Pazzianotto-Forti et al., 2020). Furthermore, an increase in fat-free body mass mediated by PA could be favorable to energy metabolism and the synthesis of anti-inflammatory cytokines (Monteiro et al., 2015; Saeidi et al., 2020). Further, obesity is well-known to decrease physical fitness and academic performance (Ortega et al., 2008; Garber et al., 2011; Wu et al., 2017). Several factors were suggested to explain the impact of obesity on reduced physical fitness. These include a lower level of PA in obese children compared to non-obese peers (Pazzianotto-Forti et al., 2020), and therefore, less opportunity to develop motor skills which might further restrict participation and promote muscular deconditioning (Knöpfli et al., 2008; Thivel, 2011). In addition, compared to non-obese peers, obese youth tend to abstain from weight-bearing actions (e.g., walking, running) due to the substantial costs of energy associated with such activities. This could lead to poor musculoskeletal and cardiorespiratory fitness (Shultz et al., 2009). Finally, it has been considered that obesity-related fitness impairments were caused by neuromuscular dysfunction due to metabolic imbalance (Wearing et al., 2006).

Accordingly, adequate intervention programs are needed to counteract energy imbalance, promote energy expenditure, and fight against youth overweight and obesity (Zouhal et al., 2020). While aerobic exercise acutely increases energy expenditure, strength training has the potential to increase the resting energy metabolism through increased muscle mass in pubertal youth and improved neural activation in pre- and pubertal youth (Nakhostin-roohi and Branch, 2018). The higher body mass of obese youth can be associated with greater muscle size and thickness compared to non-obese youth. Therefore, the combination of endurance and strength training (i.e., concurrent training) could be recommended to maintain or increase muscle mass and improve neuromuscular function while diminishing fat mass. However, until now, few studies on concurrent training were conducted with obese youth.

A dosage of 60–90 min PA per day with primarily aerobic exercises and at least 3 times per week of muscle-strengthening exercises afford appropriate sequencing of endurance and strength training to avoid interference effects (Silva, 2019). Interference occurs when strength and endurance stimuli both target peripheral (i.e., muscular) adaptations (e.g., hypertrophy *vs*. muscle capillarization). From a physiological point of view, interference effects are primarily encountered in adolescents and adults but not in pre-pubertal children because biological maturation during and after puberty enables the circulation of anabolic hormones, which is a prerequisite for training-induced muscle hypertrophy (Gäbler et al., 2018). Moreover, Vechin et al. (2021) postulated that concurrent training using high-intensity interval training (HIIT) to improve aerobic capacity minimizes chances of producing interference effects of HIIT and resistance training with regards to mitochondrial growth and muscle hypertrophy. Recently, a systematic review and meta-analysis aggregated findings of 15 studies on the effects of concurrent strength and endurance training on physical fitness and athletic performance in youth. The authors reported that concurrent training is more effective than single-mode endurance or strength training in improving components of physical fitness and athletic performance in youth (Gäbler et al., 2018). In addition, García-Hermoso et al. (2018) conducted a meta-analysis with data from 12 studies on the effects of concurrent training on body composition and metabolic markers in obese and overweight youth aged 6–18 years. The authors specifically included the results of studies that compared a concurrent training group with an aerobic training group, and found that additional strength training had small to intermediate effects on body composition, low-density lipoprotein cholesterol, and adiponectin concentrations, suggesting an improved metabolism.

On the other hand, physical fitness has proven to be an important predictor of mortality risk independent of BMI (Barry et al., 2014; Pazzianotto-Forti et al., 2020). A recent meta-analysis carried out by Barry et al. (2014) indicated that healthy weight people but with low physical fitness are two-fold more at risk of mortality than people who are over-weight and obese and have good physical fitness, which illustrate the paradox of obesity. Good physical fitness minimizes the risk of morbidity and death compared to poor physical fitness despite extra body weight. This finding highlights the necessity not just of decreasing weight but of designing programs to improve physical health (Ortega et al., 2016, 2018). Furthermore, physical fitness can be regarded as an integrated measure of most, if not all, of the body functions involved in daily PA and/or physical exercise (skeletomuscular, cardiorespiratory, hematocirculatory, psychoneurological, and endocrine–metabolic). As a result, when physical fitness is evaluated, the functional status of all of these systems is also assessed. This is why physical fitness is currently considered as one of the most important health markers, as well as a predictor of mortality and morbidity for cardiovascular disease (CVD) and all causes (Ortega et al., 2008).

Taken together, these findings indicate that concurrent training could be more effective than single-mode aerobic training in controlling body weight, body fat percentage, and reducing the risks of cardiac and metabolic diseases. Currently, it is unresolved in the literature if the additional benefits of combined or multimodal exercise types such as concurrent training are simply due to an increased exercise volume compared with single-mode exercise regimes or whether training contents (e.g., combined resistance and aerobic training versus single-mode resistance training) are responsible for the observed effects. In addition, there is a lack of studies which examined this research question in obese youth (Schroeder et al., 2019).

Therefore, the purpose of the present study was to compare the effect of three training modalities matched for volume, i.e., high-intensity intermittent training only (HIIT), resistance training only (RT), and combined HIIT and RT (i.e., concurrent training or CT), on body composition and selected components of physical fitness in sedentary youth with obesity. Based on the relevant literature (Sigal et al., 2014; Alberga et al., 2015; Racil et al., 2016), we hypothesized that CT would lead to greater improvements in body composition and physical fitness in obese youth compared to single-mode RT or HIIT.

## 2 Materials and Methods

### 2.1 Participants

A total of 73 sedentary adolescents classified as obese aged 12–14 years were recruited from four public secondary schools of the same urban region. Participants were categorized as obese according to the World Health Organization’s (WHO) child growth standards for age, sex, and BMI (BMI >97th percentile). Accordingly, youth aged 5–19 years are classified as obese if the BMI was >97th percentile (McCarthy et al., 2006; De Onis et al., 2007). This procedure was applied in the current study. ‬‬‬‬‬‬‬‬‬‬‬‬‬‬‬‬‬‬‬‬‬‬‬‬‬‬‬‬‬‬‬‬‬‬‬‬‬‬‬‬‬‬‬‬‬‬‬‬‬‬‬‬‬‬‬‬‬‬‬‬‬‬‬‬‬‬‬‬‬‬‬‬‬‬‬‬‬‬‬‬Over the course of this study, the participants maintained their normal level of PA (e.g., physical education classes) without any additional after-school physical activities. None of the obese individuals participated in a specific dietary program with the intention to lose body mass.‬‬‬‬‬‬‬‬‬‬‬‬‬‬‬‬‬‬‬‬‬‬‬‬‬‬‬‬‬‬‬‬‬‬‬‬‬‬‬‬

**FIGURE 1:**
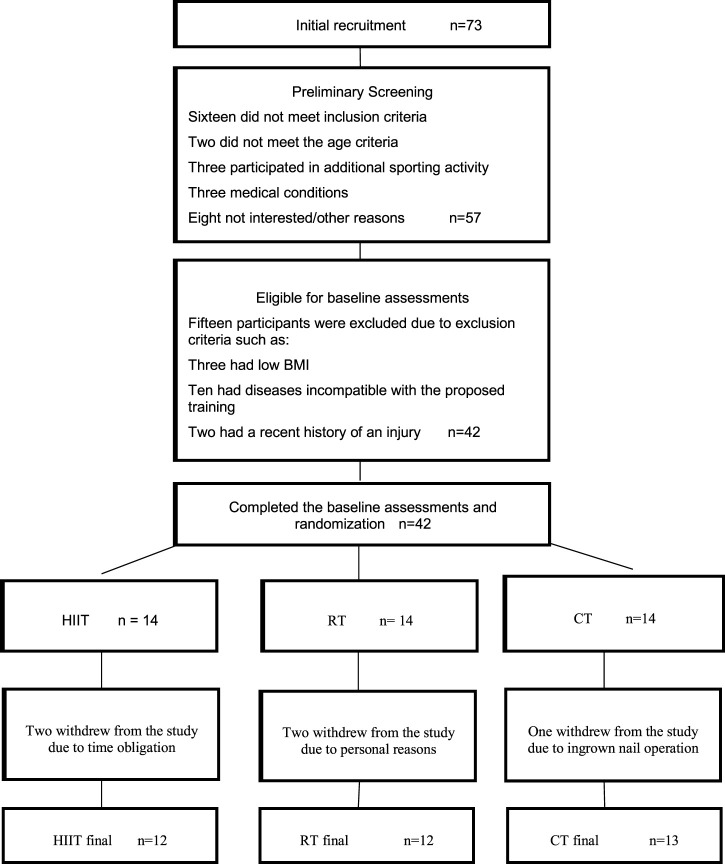
Flow diagram of the study program. HIIT, High-intensity intermittent training group; RT, Resistance training group; CT, Concurrent training group; BMI, Body mass index.

Prior to all activities, participants underwent comprehensive medical screening, conducted by a sports medicine physician at the National Center of Medicine and Science in Sports (NCMSS), to determine their eligibility for this study. The medical screening was done by the clinical physician, and included medical history, physical examination, maturity status assessment, diet, eating disorders, and socioeconomic status. Out of 73 participants, 42 were found eligible for randomization. Due to dropouts, only 37 (14 females and 23 males; HIIT, *n* = 12; RT, *n* = 12; and CT, *n* = 13) completed all phases of the intervention. The flow diagram of the study program is displayed in Figure 1.

A minimum sample size of 36 was estimated from an a priori statistical power analysis using G*Power (Version 3.1, University of Düsseldorf, Germany) (Faul et al., 2007). The analysis revealed that this sample size would be sufficient to achieve medium-sized group-by-time interaction effects. The power analysis was computed with an assumed power of 0.90, an alpha level of 0.05, and a moderate ES (Cohen’s d) based on the outcome (i.e., % body fat) of a study with similar study design (Racil et al., 2015).‬‬‬‬‬‬‬‬‬‬‬‬‬‬‬ Participants of this study were of similar medium socio-economic background. Participants’ anthropometric characteristics are presented in Table 1. Over the course of this study the participants maintained their normal level of PA (e.g., physical education classes) without any additional after-school physical activities. The International Physical Activity Questionnaire (IPAQ) adapted for adolescents was used to identify the levels of PA before and after the intervention and to determine whether the participants had already engaged in other moderate/vigorous physical activities before starting the program (Guedes et al., 2005). None of the participants was enrolled in a specific dietary program with the intention to lose body mass.‬‬‬‬‬‬‬‬‬‬‬‬‬‬‬‬‬‬‬‬‬‬‬‬‬‬‬‬‬‬‬‬‬‬‬‬‬‬‬‬‬‬‬‬‬‬‬‬‬‬ Parents or legal representatives read and signed an in-formed consent in which the procedures, risks, and benefits of the study were explained. In addition, the participating adolescents gave their written assent before any testing was conducted. This study was conducted in accordance with the Declaration of Helsinki and the protocol was fully approved by the local ethics committee of the CNMSS of Tunis (Number LR09SEP01) before the commencement of the measurements.

**TABLE 1:** Baseline anthropometric characteristics of the study participants.

	HIIT (*n* = 12)	RT (*n* = 12)	CT (*n* = 13)
Age (years)	12.8 ± 0.9	12.7 ± 0.9	13.2 ± 0.9
Body mass (kg)	75.7 ± 10.5	67.5 ± 11.3	96.7 ± 13.7^a^
Body Height (m)	1.60 ± 0.08	1.56 ± 0.11	1.63 ± 0.07
BMI (kg/m2)	29.7 ± 2.7	27.5 ± 2.5	36.2 ± 3.5^a^

Values are presented as mean ± SD. BMI, body mass index; HIIT, High-intensity intermittent training; RT, Resistance training; CT, Concurrent training.

a Significantly different from HIIT and RT (*p* < 0.01).

### 2.2 Study Design

The present study was designed so that the participants were randomly assigned to one of three training groups (allocation ratio 1:1:1). Experimental group 1 underwent aerobic training, i.e., high-intensity interval training (HIIT), group 2; Resistance Training (RT) and group 3; a combination of HIIT and RT (i.e., concurrent training or CT). Training volume was similar between groups, with the duration of training being time-matched for the three groups. Intra-session active work duration and weekly training load were similar between groups. We chose to match the training regimens based on session time and session rating of perceived exertion (s-RPE) (Foster et al., 2001), which was assessed at 10 min post-exercise, given that participants were sedentary at the beginning of the study (Donges et al., 2013). Mean session RPE score was obtained for each group and a mean RPE score per week/group was calculated. Training was conducted over 9 weeks at a frequency of three sessions per week. Before and after training, tests were applied to assess body com-position and physical fitness. At post-training, testing was performed within 48–72 h after the last training session to ensure optimal recovery (Monteiro et al., 2015). A 2-week familiarization period was included prior to the start of the study to allow participants to become acquainted with exercise and test procedures.

### 2.3 Procedure

#### 2.3.1 Dietary Counselling

All participants received the same dietary counselling at baseline and after 1 month by a dietitian to minimize dietary variability among groups and promote healthy eating. This counselling was focused on changing the quality of the diet without changing the total energy intake to avoid weight loss. Energy intake was assessed at baseline and after the intervention through an interview using a 3-days food record (two weekdays and one weekend day). Dietary records were analysed for energy intake (kcal) using the professional Nutri Pro 7 program (Nutri Pro 7 software, CERDEN, Brussels, Belgium).

#### 2.3.2 Anthropometric Measures and Body Composition

Pre- and post-training anthropometrics was recorded in a climate-controlled temperature setting by an experienced examiner on two consecutive days. The participants were barefooted and lightly clothed. Anthropometrics such as body height (BH), body mass (BM), BMI, and waist circumference (WC) were taken on the first day. On day two, body composition of the participants was measured using dual energy X-ray absorptiometry (DEXA). BM was measured using a digital scale (Seca Instruments Ltd., Hamburg, Germany). Body height (BH) was determined with a wall-mounted stadiometer (Holtain Ltd., Crymych, United Kingdom). WC was measured at the midpoint between the lower rib and the iliac crest at the end of a normal exhalation using a tape measure in accordance with standard anthropometric protocols (Norton, 2018). Body composition such as body fat (BF in kg), fat-free mass (FFM in kg), percentage body fat (%BF) was assessed *via* DEXA scanner (Lunar Prodigy Advance–IQ version 4.7e pediatric software).

#### 2.3.3 Physical Fitness Tests

##### 2.3.3 1 Counter Movement Jump

The CMJ required the participants to flex the knees as quickly as possible from an up-right standing position to a self-selected depth, followed immediately by a vertical jump. Participants were instructed to jump as high as possible. The CMJ measurement was ac-quired using an Optojump^®^ system (Micro Gate Optojump Next, Italy). During CMJ performance, participants were instructed to place their hands on the hips to minimize lateral and horizontal displacements during the task, to prevent any influence of arm movements on the vertical jumps (Chaouachi et al., 2014). Participants were educated at the take-off from the jump to keep their knees fully extended and their ankles in a plantar flexed position. They were instructed to land in the same position and location to minimize horizontal displacement and flight time. Three trials were performed with a 2-min rest in between the trials and the best jump height was used for further analysis.

##### 2.3.3 2 Medicine Ball Throw

The medicine ball throw test was used to assess the upper-body strength (Van Den Tillaar and Mário, 2013). The participant sat on the ground with their legs in front of them while keeping both hands on their chest and their back against a wall. They were then in-structed to throw a medicine ball of 3 kg with maximal effort as far as possible in front of them. The examiner verified the correct throwing technique during the study. The distance from the wall to where the ball landed was recorded and the best result of three throws was used for statistical analysis.

##### 2.3.3 3 Maximal Voluntary Isometric Contraction Handgrip Test

Handgrip strength was measured using a calibrated hand dynamometer (Takei, Tokyo, Japan). Participants stood comfortably with the arm abducted at approximately 45°. The dynamometer was held freely without support and did not touch the subject’s trunk, with constant extension of the elbow. The grip span of the dynamometer was adjusted to each participant’s hand size so that the proximal inter-phalangeal joints of the four fingers rested on one side of the handgrip and that of the thumb rested on the other side (Makhlouf et al., 2018). Participants were required to exert MVIC strength on the dynamometer. Three trials were done for each hand. The handgrip score (kg) was calculated as the average of the left and right and then expressed per kilogram of body mass (hand-grip/body mass) (Agostinis-Sobrinho et al., 2017).

##### 2.3.3 4 Maximal Voluntary Isometric Contraction of the Knee Extensors

MVIC of the knee extensors was measured using a calibrated hand-held dynamometer (Microfet 2, Hogan Health Industries Inc., Draper, UT, United States). The dynamometer had a built-in load cell with a digital display. The hand-held dynamometer was placed perpendicular to the anterior aspect of the tibia, just proximal of the medial malleolus. Participants were seated on a leg extension machine, with both feet off the ground and hips and knees flexed at 90°. The lever arm of the leg extension machine was fixed at 100° with the dynamometer attached to it (Makhlouf et al., 2018). Participants were instructed to exert the highest force as fast and hard as possible against the dynamometer for a period of 3–5 s. Three consecutive trials separated by a 1-min rest were completed for both legs and the highest values were recorded for further analysis.

##### 2.3.3 5 Maximal Voluntary Isometric Contraction of the Back Extensors

Maximal isometric back extensor strength was measured in kilograms with a back and leg dynamometer (Takei, Tokyo, Japan) in the same manner as previously described (Hammami et al., 2016). Participants stood shoulder-width apart on the dynamometer foot stand and gripped the handle bar positioned across the thigh. The chain length on the dynamometer was adjusted so that the legs were straight and the hips were flexed at a 30° angle to position the bar at the level of the patella. Participants were then asked to straighten their backs (i.e., stand upright) without bending their knees and lifted the dynamometer chain, with the pulling force applied on the handle, pulling upwards as strongly as possible. Participants completed three trials; the highest score being recorded as the measurement of maximal back force under isometric conditions. A 30-s rest interval was provided between trials.

##### 2.3.3 6 Speed

A 10-m sprint and a 30-m maximal sprint test were used to assess acceleration and maximal running speed as previously described (Makhlouf et al., 2018). The 10-m sprint involved sprinting as fast as possible from a stationary standing start position just behind the first timing gate for 10-m. The 30-m maximal sprint involved sprinting as fast as possible from a moving start. Participants were placed 20 cm behind the starting line and in-structed to run as fast as they could along a 30-m distance. Time was automatically recorded using photocell gates 0.4 m above the ground (Brower Timing Systems, Salt Lake City, Utah, USA, precision 0.01 s). The fastest run for each test was selected for analysis.

##### 2.3.3 7 Agility

Participants’ agility was evaluated with the 4 × 9-m shuttle run test. The participant started from a standing position with their feet behind the start line. To successfully complete each 9-m segment, participants were required to cross the start and finish lines, with one foot. The timer started as the participant passed the first timing gate at the start line and stopped passing the same timing gate after the final 9-m. The best time of two consecutive trials was recorded for the statistical analysis. A 2-min rest was provided between trials.

##### 2.3.3 8 Six-Minute Walking Test (6MWT-distance; 6MWT-VO_2max_)

To assess the submaximal aerobic functional capacities of the participants, the 6-min walking test (6 MWT) was used. This protocol has been validated for obese children (Elloumi et al., 2011). This test was conducted on a flat surface in a 30-m long covered corridor marked every 3-m according to the recommendations of the American Thoracic Society (ATS, 2002). Participants were instructed to walk the longest distance possible at their own pace during the allotted time. Individuals were allowed to stop and rest during the test, but were instructed to resume walking as soon as they felt capable of doing so. Standardized encouragements (for example, “Keep going”, “You are doing well”) and announcement of time remaining were given to the participants. Walking performance was quantified by using the 6 MWT-distance. A prediction equation was developed to estimate VO_2max_, using the distance walked in 6 MWT and BMI (Vanhelst et al., 2013). The prediction equation is the following:

$${\text{VO}}_{2\text{max}}\left(\text{ml}{\text{.kg}}^{-1}{\text{.min}}^{-1}\right)=26\text{.}9+\text{0}\text{.}014\text{×}6\text{MWT}\left(\text{m}\right)-\text{0}\text{.}38\text{×BMI}\left(\text{kg}/{\text{m}}^{2}\right)$$

##### 2.3.3 9 Spartacus Test

Participants completed an intermittent (15–15 s), progressive, and maximal run test to evaluate the intermittent peak velocity (Vpeak) in order to prescribe and monitor training intensities. The Spartacus intermittent test is considered as a relevant field test to indirectly assess the global level of aerobic capacity of obese adolescents (Rey et al., 2013, 2016). A rectangle of 750 m^2^ (75 × 10) was created with different marks set at regular intervals, which represent the different speeds (from 1.94 to 5.0 m/s). Each stage lasts 3 minutes with 1 min 30 s of running and 1 min 30 s of passive recovery. During the 3 minutes (for each stage/speed), participants had 15 s to reach the corresponding mark and then 15 s of passive rest. The first stage was set at 1.94 m/s and each following stage in-creasing by 0.3 m/s every 3 minutes. The participants were stopped by the examiner when they were not able to keep up with the beeps at the stage marks twice in succession. The adolescents ran in groups of no more than five participants (Fillon et al., 2020). Rating of perceived exertion was expressed by each participant immediately after running test on a 6–20 scale (Groslambert et al., 2001). Running performance was evaluated through the total run duration and the peak velocity value (Vpeak), which considered the last fully completed stage and additional run time.

### 2.4 Training Programs

After testing, volunteers were randomly assigned to three training groups and began to exercise 3 days per week over 9-weeks in the same sport facilities. All groups had the same overall exercise duration (time-matched). Training groups consisted of HIIT (*n* = 12), RT (*n* = 12), and CT (*n* = 13). All training sessions (HIIT, RT and CT) started with a standardized warm-up (e.g., 5 min of jogging, 5 min of dynamic stretching, 5 min of balance exercises and 5 sprints over 20-m) and ended with a cool-down for 5 min.

High-Intensity Interval Training (HIIT). The HIIT program consisted of 12 short-interval, intermittent high intensity exercises bouts (10-s activity, 10-s passive rest). Intensity was individualized ranging from 80 to 110% of the participant’s Vpeak according to their Vpeak determined at the end of the 15-s/15-s Spartacus intermittent run test (Thivel et al., 2017). For example, a subject with a Vpeak of 9 km/h had to run a 25-m distance in 10-s at 100% of Vpeak. Afterwards, they had a 10-s passive recovery followed by another 10-s run back to where they had started. Participants performed 3 or 4 sets with an effective work time of 6 or 8 min per session, respectively. A 3-min passive recovery was allowed between each set. The program was inspired from Baquet et al. (2002). Running times were controlled using a sport beeper producing a sound every 10-s. The training program is outlined in Table 2.

**TABLE 2:** High-intensity intermittent training (HIIT).

Week (n°)	Sets × (repetitions × duration)	%V_peak_	Recovery Between Sets (min)
1	3 × (12 × 10 s)	80	3
2	3 × (12 × 10 s)	90	3
3	4 × (12 × 10 s)	80	3
4	4 × (12 × 10 s)	90	3
5	3 × (12 × 10 s)	100	3
6	3 × (12 × 10 s)	110	3
7	4 × (12 × 10 s)	100	3
8	4 × (12 × 10 s)	110	3
9	3 × (12 × 10 s)	100	3

V_peak_: Intermittent peak velocity measured from the Spartacus test.

Resistance Training. Resistance training was conducted using elastic bands (Thera-band; The Hygenic, Co., Akron, OH, USA). This method of resistance loading was selected for its ease of use and its suitability for children due to its low cost, efficacy for strength gains in different population profiles (Lopes et al., 2019) and benefits for preventing obesity (Liao et al., 2017). Each session consisted of six single and multiple-joint resistance exercises including squats, chest presses, shoulder presses, rowing exercise, straight leg hip extension, and biceps curls against a Theraband. These exercises were alternated each week with chest flyes or push-ups, shoulder flyes, chin-ups, pull-ups either assisted or eccentric (jump up and lower the body slowly), and overhead triceps extensions. The participants started off with a band colour with which they could perform the 10-repetition maximum (RM). The degree of resistance load was manipulated using bands with varying degrees of difficulty according to their colour code (e.g., yellow, red, green, and blue in order of increasing resistance provided) once the participants were able to perform more than 10 repetitions, the resistance of the exercise was progressed by upgrading to the next colour of Theraband according to the manufacturer’s guidelines. The training commenced from the upper-body and progressed to the lower-body, in which they completed compound multi-joint exercises prior to isolation exercises (Donges et al., 2013). The program followed the same periodized training design as HIIT with youth initially completing 3 sets of 10 RM for all exercises. Each exercise set took approximately 20-s to complete (1:1 tempo [1-s eccentric, 1-s concentric]) (Magnani Branco et al., 2020) with a total work time of 60-s per exercise. An exercise session was completed in 6 or 8 min based on the amount of sets they had to execute (3 or 4 sets). All exercise sessions were supervised with a maximum participant-to-staff ratio of 6:1.

Concurrent Training. CT consisted of a combination of HIIT and RT that was time-matched by having half of HIIT and half of the RT volume. Thus, participants in the CT group completed the same work volume over the course of the study as HIIT and RT participants. Participants performed similar exercises on the same equipment, with identical relative intensity, and in the same order as RT and HIIT. 1.5 to 2 sets × 10 maximum repetitions of each RT exercise were completed, and was followed by 1.5–2 sets × 12 bouts of HIIT exercise. The second half set (5 repetitions for RT and 6 bouts for HIIT) was completed at the same absolute resistance/intensity as the first set (10 repetitions for RT/12 bouts for HIIT) (Donges et al., 2013).

### 2.5 Statistical Analyses

Data are presented as means and standard deviations (SD) in figures, text and tables. Test-retest reliability of the variables was assessed using Cronbach’s model of intra-class-correlation coefficients (ICC _(3,1)_)_._ ICCs were presented with 95% confidence intervals, SEMs and coefficient of variation (CV) according to the method of Hopkins (Hopkins et al., 2009). ICC can be classified as very small (<0.1), low (0.1–0.3), moderate (0.3–0.5), high (0.5–0.7), very high (0.7–0.9), and nearly perfect (>0.9). Normality was assessed and confirmed using the Kolmogorov-Smirnov test. Data were then analysed using a 3 (groups: HIIT, RT, CT) × 2 (time: pre, post) ANOVA with repeated measures. If group-by-time interactions reached the level of significance, group-specific and Bonferroni adjusted post-hoc tests (i.e., paired t-tests) were computed to identify the comparisons that were statistically significant. Additionally, the classification of effect sizes (ES) was determined from ANOVA output by converting partial eta-squared to Cohen’s d (Cohen, 1988). Moreover, within-group Cohen’s d effect sizes were computed using the following equation: effect size = (mean_post–mean_pre)/pooled SD. According to Cohen, effect sizes can be classified as small (0.00 ≤ d ≤ 0.49), medium (0.50 ≤ d ≤ 0.79), and large (d ≥ 0.80). For the varying groups, i.e., HIIT, RT, and CT, contrast analyses (Williams and Abdi, 2010) were carried out to specifically test the hypothesis that the CT-group would lead to greater changes in the outcome measures than the single-mode HIIT and RT groups (coded as -0.667, 0.333, and 0.333, respectively). This approach yields a comparison of one (or more) condition(s) *vs*. the grand mean of the specified contrasts. Indeed, post-hoc analyses, while useful, do not yield sufficient insight into multiple levels or detailing patterns in response, whereas contrast analyses allow researchers to test theory-driven expectations directly against empirically derived group or cell means (Williams and Abdi, 2010). The alpha level of significance was set at *p* ≤ 0.05. All data analyses were performed using the statistical package for social sciences (IBM SPSS Inc., Chicago, IL, version. 25.0).

## 3 Results

Finally, out of 73 screened participants, 42 were found eligible for randomization. Due to dropouts, only 37 (14 females and 23 males) completed the study according to the study design and methodology. Thus, the final analyses were performed on 37 adolescents (12 HIIT, 12 RT, and 13 concurrent, respectively). All participants received treatments as allocated.

### 3.1 Inquiry Data

Analysis of variance showed no significant time effect for the total energy intake (EI) (F = 0.13; *p* > 0.72; d = 0.06) within HIIT, RT and CT training groups after 9 weeks of training. In addition, no significant group × time interaction effects were found for EI (F = 0.005, *p* > 0.99, d = 0).

### 3.2 Reliability Measures

Table 3 displays the test-retest reliability analyses for all performed tests. ICCs showed excellent reliability for all dependent variables. ICC-values ranged from 0.95 to 0.99, with a standard error of measurement (SEM) from 0.02 to 2.36 and an acceptable coefficient of variation (CV< 5%). Furthermore, paired t-tests showed no significant differences (*p* > 0.05) between the scores recorded during the two trials for all measured variables.

**TABLE 3:** Test-retest reliability of physical fitness tests.

	Test mean ± SD	Retest mean ± SD	Mean difference (±SD)	ICC [95% CI]	SEM	CV%
10-m sprint (s)	2.66 ± 0.29	2.65 ± 0.28	0.01 ± 0.08	0.95 [0.957–0.989]	0.02	2.2
30-m sprint (s)	6.81 ± 0.95	6.84 ± 0.97	−0.03 ± 0.15	0.98 [0.988–0.997]	0.03	1.5
Agility (s)	12.42 ± 1.03	12.43 ± 1.03	−0.01 ± 0.16	0.98 [0.988–0.997]	0.04	0.9
MVIC back extensor strength (kg)	68.1 ± 22.2	66.9 ± 22.2	1.3 ± 2.8	0.99 [0.993–0.998]	0.61	3.1
MVIC Right KE force (N)	291.0 ± 86.9	290.2 ± 84.8	0.7 ± 9.8	0.99 [0.994–0.998]	2.19	2.4
MVIC Left KE force (N)	279.6 ± 79.2	280.4 ± 82.6	−0.8 ± 10.6	0.99 [0.992–0.998]	2.36	2.6
CMJ (cm)	11.9 ± 4.4	11.8 ± 4.0	0.1 ± 0.7	0.98 [0.986–0.996]	0.16	4.3
MB throw distance (m)	2.9 ± 0.6	2.8 ± 0.7	0.03 ± 0.08	0.99 [0.993–0.998]	0.02	2.0
6MWT-distance (m)	621.6 ± 72.2	619.5 ± 72.6	2.1 ± 8.4	0.99 [0.993–0.998]	1.87	1.0

MVIC, maximum voluntary isometric contraction; KE, knee extensors; CMJ, Countermovement jump; MB, medicine ball; 6MWT, 6 minute walking test; ICC, Intra-class coefficient; CV, Coefficient of variation; SEM, Standard error measurement.

### 3.3 Anthropometric Characteristics and Body Composition

Analyses of variance showed significant time effects for all dependent variables (*p* < 0.001 for all, d = 0.70–1.62). The results also revealed significant group × time interaction effects for BM (F = 21.87, *p* = 0.001, d = 1.07), BF (F = 9.60, *p* = 0.002, d = 0.67), BMI (F = 10.40, *p* = 0.001, d = 0.72) and WC (F = 6.72, *p* = 0.004, d = 0.63). BM, BF (kg) and BMI decreased significantly more in the CT group (-4.5%, -9.0% and -5.7%, respectively) compared to the HIIT group (-1.6%, -6.7% and –2.6%, respectively) and the RT group (-1.8%, -6.0% and -3.1%, respectively). In addition, WC decreased significantly more in the CT and RT groups (-4.4 and -4.8%, respectively) compared to the HIIT group (-1.3%) (see Table 4).

**TABLE 4:** Effects of 9 weeks of training on participants’ anthropometric characteristics and body composition (mean ± SD).

Variables	Group	Pre	Post	Change %	Cohen’s d	ANOVA. *p*-value (Cohen’s d)		
						Time	Group	Interaction
Body mass (kg)	HIIT	75.7 ± 10.5	74.5 ± 11.3^**^	−1.6	0.11	0.001 (1.62)	0.001 (1.03)	**0.001** (1.07)
	RT	67.5 ± 11.3	66.3 ± 11.5^**^	−1.8	0.11			
	CT	96.7 ± 13.7	92.4 ± 13.0^**^ ^a^ ^,^ ^b^	−4.5	0.31			
Body fat (%)	HIIT	49.2 ± 5.4	47.1 ± 5.0^**^	−4.2	0.39	0.001 (1.07)	0.001 (0.69)	0.472 (0.21)
	RT	44.5 ± 5.1	42.3 ± 6.7^**^	−4.9	0.43			
	CT	52.3 ± 2.3	49.2 ± 2.5^**^	−6.1	1.38			
Fat-free mass (kg)	HIIT	37.0 ± 7.8	38.3 ± 8.1^**^	3.4	0.16	0.001 (0.70)	0.01 (0.55)	0.494 (0.21)
	RT	35.8 ± 6.7	36.5 ± 6.5	1.9	0.10			
	CT	44.0 ± 7.0	45.5 ± 7.3^**^	3.4	0.21			
Body fat (kg)	HIIT	35.5 ± 5.0	33.1 ± 4.6^**^	−6.7	0.48	0.001 (0.69)	0.001 (1.35)	**0.002** (0.67)
	RT	28.7 ± 5.9	27.0 ± 6.3^**^	−6.0	0.29			
	CT	48.2 ± 7.0	43.9 ± 6.4^**^ ^a^ ^,^ ^b^	−9.0	0.62			
BMI (kg/m^2^)	HIIT	29.7 ± 2.7	28.9 ± 2.9^**^	−2.6	0.29	0.001 (1.49)	0.001 (1.27)	**0.001** (0.72)
	RT	27.5 ± 2.5	26.6 ± 2.5^**^	−3.1	0.34			
	CT	36.2 ± 3.5	34.1 ± 2.8^**^ ^a^ ^,^ ^b^	−5.7	0.58			
WC (cm)	HIIT	86.9 ± 6.8	85.8 ± 7.6	−1.3	0.16	0.001 (1.34)	0.001 (0.75)	**0.004** (0.63)
	RT	89.5 ± 7.3	85.2 ± 6.9^**^ ^a^	−4.8	0.59			
	CT	99.9 ± 7.2	95.5 ± 7.8^**^ ^a^	−4.4	0.60			

^**^ Significant difference from pre to post (*p* < 0.01). The bold values mean that Interaction Anova p-Value and Contrast p-Value are significant; given that the alpha level of significance was set at *p* ≤ 0.05.

a Significantly different from HIIT (*p* < 0.05).

b Significantly different from RT (*p* < 0.05).

c Significantly different from CT (*p* < 0.05); HIIT, High-intensity intermittent training group; RT, resistance training group; CT, concurrent training group; BMI, body mass index; WC, waist circumference.

### 3.4 Physical Fitness Outcomes

Analyses of variance showed significant time effects for all assessed components of physical fitness (*p* < 0.001 for all, d = 0.99–2.94) (see Tables 5–7). Significant group-by- time interaction effects were also found for the MB throw-distance (F = 4.63, *p* = 0.03, d = 0.56), handgrip strength/body mass (F = 3.98, *p* = 0.03, d = 0.49), CMJ (F = 5.38, *p* = 0.01, d = 0.56), 6 MWT-VO_2max_ (F = 7.81, *p* = 0.001, d = 0.68), and 6 MWT-distance (F = 3.57, *p* = 0.004, d = 0.46). More specifically, post-hoc tests showed greater improvements in the CT group compared to HIIT group for the MB throw distance (+20.2%, d = 0.80 *vs*. +13.6%, d = 0.68, respectively) and 6 MWT-VO_2max_ (+12.0%, d = 1.12 *vs*. +7.5%, d = 1.15, respectively) and 6 MWT-distance (+20.1%, d = 1.36 *vs*. +17.3%, d = 1.50, respectively). In addition, the CT group displayed significantly greater improvements than the RT group in 6 MWT-VO_2max_ (+12.0%, d = 1.12 *vs*. +4.9%, d = 0.76) and 6 MWT-distance (+20.1%, d = 1.36 *vs*. +10.4%, d = 0.79). On the other hand, the RT group obtained significantly greater improvements than HIIT and CT groups in handgrip strength/body mass (+28.6%, d = 0.90 *vs*. +10.2%, d = 0.43 and +17.1%, d = 0.48, respectively) and CMJ (+42.8%, d = 0.89 *vs*. +23.3%, d = 0.76 and +20.8%, d = 0.76, respectively).

**TABLE 5:** Effects of 9 weeks of training on sprint and agility tests (mean ± SD).

Variables	Group	Pre	Post	Change %	Cohen’s d	ANOVA *p*-value (Cohen’s d)		
						Time	Group	Interaction
10-m sprint (s)	HIIT	2.58 ± 0.22	2.44 ± 0.24**	−5.6	0.64	0.001 (1.22)	0.552 (0.22)	0.874 (0.10)
	RT	2.64 ± 0.33	2.46 ± 0.28**	−6.9	0.55			
	CT	2.66 ± 0.28	2.48 ± 0.27**	−6.7	0.64			
30-m sprint (s)	HIIT	6.56 ± 0.85	6.31 ± 0.73**	−3.7	0.29	0.001 (0.99)	0.437 (0.26)	0.637 (0.19)
	RT	6.77 ± 0.93	6.36 ± 0.75**	−6.1	0.44			
	CT	6.99 ± 1.05	6.63 ± 0.79**	−5.1	0.34			
Agility (s)	HIIT	12.41 ± 1.12	11.67 ± 0.95**	−6.0	0.66	0.001 (1.51)	0.462 (0.25)	0.995 (0.003)
	RT	12.19 ± 1.02	11.41 ± 0.86**	−6.4	0.76			
	CT	12.49 ± 0.93	11.70 ± 0.83**	−6.3	0.84			

^**^ Significant difference from pre to post (*p* < 0.01).

a Significantly different from HIIT (*p* < 0.05).

b Significantly different from RT (*p* < 0.05).

c Significantly different from CT (*p* < 0.05); HIIT, High-intensity intermittent training group; RT, resistance training group; CT, Concurrent training group.

**TABLE 6:** Effects of 9 weeks of training on strength and jumping tests (mean ± SD).

Variables	Group	Pre	Post	Change %	Cohen’s *d*	ANOVA *p*-value (Cohen’s *d*)		
						Time	Group	Interaction
MVIC back extensor strength (kg)	HIIT	67.8 ± 18.6	96.3 ± 24.0**	42.0	1.53	0.001 (1.67)	0.377 (0.24)	0.417 (0.21)
	RT	60.3 ± 22.1	89.6 ± 25.0**	48.6	1.32			
	CT	77.1 ± 24.9	99.0 ± 30.5**	28.4	0.88			
Medicine ball throw distance (m)	HIIT	2.9 ± 0.6	3.3 ± 0.7**	13.6	0.68	0.001 (2.94)	0.046 (0.53)	**0.035** (0.56)
	RT	2.7 ± 0.5	3.2 ± 0.5**	17.4	0.95			
	CT	3.0 ± 0.8	3.7 ± 0.8**^,^ ^a^	20.2	0.80			
Handgrip strength/body mass	HIIT	0.7 ± 0.2	0.7 ± 0.2**	10.2	0.43	0.001 (1.24)	0.571 (0.18)	**0.028** (0.49)
	RT	0.6 ± 0.2	0.8 ± 0.3**^,^ ^a^ ^,^ ^c^	28.6	0.90			
	CT	0.6 ± 0.2	0.7 ± 0.2**	17.1	0.48			
MVIC Right KE force (N)	HIIT	301.8 ± 115.5	386.3 ± 130.4**	28.0	0.73	0.001 (2.29)	0.93 (0.07)	0.06 (0.48)
	RT	274.1 ± 77.0	412.7 ± 96.3**^,^ ^a^	50.5	1.80			
	CT	278.2 ± 63.9	380.6 ± 108.5**	36.8	1.60			
MVIC Left KE force (N)	HIIT	285.9 ± 84.7	381.6 ± 134.6**	33.5	1.13	0.001 (1.24)	0.401 (0.23)	0.862 (0.10)
	RT	246.3 ± 57.8	335.3 ± 91.2**	36.1	1.54			
	CT	287.5 ± 60.9	367.1 ± 97.5**	27.7	1.31			
CMJ (cm)	HIIT	12.5 ± 3.8	15.38 ± 3.92**	23.3	0.76	0.001 (1.72)	0.76 (0.27)	**0.009** (0.56)
	RT	12.0 ± 5.7	17.1 ± 5.6**^,^ ^a^ ^,^ ^c^	42.8	0.89			
	CT	12.0 ± 3.3	14.5 ± 3.0**	20.8	0.76			

** Significant difference from pre to post (*p* < 0.01).

a Significantly different from HIIT (*p* < 0.05).

b Significantly different from RT (*p* < 0.05).

c Significantly different from CT (*p* < 0.05); HIIT, High-intensity intermittent training group; RT, resistance training group; CT, concurrent training group; KE, knee extensors; N, newton; MVIC, maximal voluntary isometric contraction; CMJ, countermovement jump. The bold values are mean that Interaction Anova p-Value and Contrast p-Value are significant; given that the alpha level of significance was set at *p* ≤ 0.05.

**TABLE 7:** Effects of 9 weeks of training on the six-minute walking test (mean ± SD).

Variables	Group	Pre	Post	Change %	Cohen’s *d*	ANOVA *p*-value (Cohen’s *d*)		
						Time	Group	Interaction
6MWT-VO_2max_ (ml.kg^−1^ min^−1^)	HIIT	24.2 ± 1.4	25.8 ± 1.3^**^	7.5	1.15	0.001 (2.27)	0.001 (0.75)	0.011 (0.68)
	RT	24.9 ± 1.7	26.1 ± 1.7^**^	4.9	0.76			
	CT	21.7 ± 2.1	24.1 ± 1.4^**^ ^a^ ^,^ ^b^	12.0	1.12			
6MWT-distance (m)	HIIT	612.7 ± 60.9	704.2 ± 53.6^**^	17.3	1.50	0.001 (2.27)	0.447 (0.22)	0.004 (0.46)
	RT	600.2 ± 85.5	667.3 ± 87.7^**^	10.4	0.79			
	CT	613.5 ± 81.7	724.8 ± 63.3^**^ ^a^ ^,^ ^b^	20.1	1.36			

^**^ Significant difference from pre to post (*p* < 0.01).

a Significantly different from HIIT (*p* < 0.05).

b Significantly different from RT (*p* < 0.05).

c Significantly different from CT (*p* < 0.05); HIIT, High-intensity intermittent training group; RT, resistance training group; CT, concurrent training group; 6MWT, six minutes walking test.

### 3.5 Contrast Analyses

Further analyses using the method of contrasts (CT *vs*. HIIT+ and RT) confirmed greater decrements in BM, BF (in kg), BMI, and WC in the CT group compared to single-mode HIIT and RT groups (*p* < 0.036 to 0.001) (see Table 8). In addition, contrast analyses highlighted significantly greater improvements for the MB throw distance, 6 MWT-VO_2max_ and 6 MWT-distance in the CT group compared to HIIT and RT groups (*p* < 0.028 to 0.001). Conversely, we found that HIIT and RT yielded significantly greater Improvements compared to CT in CMJ (*p* < 0.028) (see Table 8).

**TABLE 8:** Contrast analyses of groups for all dependent variables.

		CT *vs*. HIIT and RT			
		Difference CT *vs*. HIIT + RT	*t*	Standard error	*p*-Value
Anthropometric variables	Body mass (kg)	−3.09	6.22	0.33	**0.001**
	Body fat (%)	−1.03	1.24	0.55	0.225
	Body fat (kg)	−2.27	3.82	0.39	**0.001**
	FFM (kg)	−0.50	0.80	0.39	0.431
	BMI (kg/m^2^)	−1.24	−3.70	0.22	**0.002**
	WC (cm)	−1.68	2.19	0.50	**0.036**
Physical fitness components	10 m sprint (s)	−0.02	0.268	0.037	0.790
	30 m sprint (s)	−0.03	0.238	0.083	0.813
	Agility (s)	0.02	0.11	0.12	0.914
	MVIC back extensor strength (kg)	6.99	1.22	3.77	0.230
	Medicine ball throw (m)	0.15	2.30	0.04	**0.028**
	Handgrip strength/body mass)	−0.03	0.88	0.02	0.386
	MVIC Right KE force (N)	50.30	1.96	16.94	0.06
	MVIC Left KE force (N)	12.78	0.50	16.88	0.621
	CMJ (cm)	−1.50	2.32	0.43	**0.028**
	6MWT-VO_2max_ (ml.kg^−1^. min^−1^)	0.92	3.79	0.16	**0.001**
	6MWT-distance (m)	31.94	2.25	9.39	**0.031**

FFM, Fat-free mass; BMI, body mass index; WC, waist circumference; MVIC, maximal voluntary isometric contraction; KE, knee extensors; CMJ, countermovement jump; 6MWT, six minutes walking test; VO_2max_, maximal O_2_ uptake. The bold values are mean that Interaction Anova p-Value and Contrast p-Value are significant; given that the alpha level of significance was set at *p* ≤ 0.05.

## 4 Discussion

To the best of authors’ knowledge, this is the first study that investigated the effects of an equal volume of three training modalities (endurance, strength and CT) on body composition and performance-related responses to 9 weeks of training in a sedentary, healthy, youth population with obesity. The most important findings were that in subjects with a BMI ≥97th percentile for age and sex, exercising in general (resistance alone, HIIT alone, CT) resulted in body mass, BMI and body fat loss and improved endurance (estimated 6 MWT-VO_2max_) but, with a more pronounced effect with CT on these four parameters than HIIT or RT, alone. Moreover, CT was found to be more efficient in MBthrow when compared to RT and HIIT, respectively. Further, the three modalities of training were also beneficial for CMJ and handgrip strength normalized to body mass, but with a lesser effect of CT. For the remaining physical fitness components (speed, agility, muscular strength of the knee and back extensors), the three modalities were found to be advantageous but without domination of one of the modalities. Consistent with our hypothesis, the present results provide evidence that CT is a promising tool for management of obesity compared to HIIT or RT training modalities.

### 4.1 Body Composition and Anthropometric Parameters

It is well established that supervised exercise intervention or regular PA induces significant reductions in body weight and fat mass as well as increase fat-free mass in obese individuals (Zouhal et al., 2020). Aerobic endurance exercise is purported to be the most effective strategy for improving anthropometric measures (Donnelly et al., 2013; Zouhal et al., 2020) in a variety of age groups (adolescents, adults and elderly) with significant decreases in body weight, body mass index (BMI), body fat and increases in free fat mass.

In the current study, the HIIT program resulted in a slight body weight loss and a small body fat loss after 9 weeks of training (ES: 0.11–0.48). These findings are consistent with previous HIIT studies demonstrating beneficial effects of intermittent exercises on body weight in overweight and obese adolescents (Racil et al., 2013; Lau et al., 2015). The magnitude of body mass loss after HIIT training alone in our study (-1.57%; ES = 0.11) was comparable to that found by Ouerghi et al. (Ouerghi et al., 2017) with obese male adolescents after 8 weeks of training (-1.62%; ES = 0.11). The HIIT group did not reduce WC, which is consistent with the meta-analysis of García-Hermoso et al. (2016) where HIIT interventions between 4 and 12 weeks were not efficient at reducing waist and hip circumferences in children and adolescents. However, our results demonstrate a significant decrease in body fat contrary to findings of the aforementioned meta-analysis showing no decrease in BM and BF after HIIT training. These last results are in accordance with those reported in a recent meta-analysis (Türk et al., 2017) concluding that HIIT is a time-efficient alternative that induces greater reductions in body fat percentage compared to traditional exercise programs in adults with obesity. These HIIT-induced body composition benefits could be partly attributed to the higher concentration of the catecholamines, which increase lipolysis in adipose tissues, and greater metabolic rate and fat expenditure resulting from HIIT compared to moderate-intensity exercise training (Zouhal et al., 2013; Zhang et al., 2017). Nevertheless, it should also be noted that some studies reported no changes in body composition after the HIIT program in sedentary people (Sasaki et al., 2014). Differences in the participants’ age, health/PA status, exercise intensity (i.e., parameters of the HIIT program), and particularly the duration of intervention may underlie the discrepancy. For example, a 4-weeks HIIT program may not be sufficient to cause significant changes in body composition in obese men in the studies by Alkahtani et al. (2013) and Sasaki et al. (2014). Thus, as suggested by Zouhal et al. (2020) in their recent review, HIIT may be an alternative to conventional exercises such as continuous exercise to treat overweight and obesity.

These findings are consistent with other studies (Schranz et al., 2013; Nakhostin-roohi and Branch, 2018) that demonstrated pronounced effect on body mass loss, BMI and body fat loss with CT. These results can mainly be explained by the increased energy expenditure from the CT, as during the 9-week period no significant caloric intake variations were identified. In addition, a meta-analysis of 12 studies by García-Hermoso et al. (2018) found that additional strength training had small to intermediate effects on body composition (ES: 0.14–0.47). O’Donoghue et al. (2021) also performed a meta-analysis comparing the efficacy of vigorous and moderate intensity aerobic exercise, high and low-to-moderate load resistance training, combined training (COM-HI and COM-LM) showing a minimal overall weight loss. They also showed that COM-HI had the highest likelihood of achieving body mass loss, which is consistent with our results. The present study showed that after 9 weeks of CT, obese youth had a greater increase in FFM although RT alone did not indicate any substantial increase. Thereby, it is possible that the training status of sedentary children and adolescents played a part in this potentiating effect. As seen by Coffey and Hawley (2017) “untrained individuals have a greater capacity to activate the molecular machinery in muscle in response to contractile activity, because any overload stimulus induces large perturbations to cellular homeostasis regardless of the mode of exercise”.

CT (HIIT and RT) mechanisms would suggest that HIIT increases oxidative activity (Tang et al., 2006) and fat oxidation (Nakhostin-roohi and Branch, 2018) due to the increase of catecholamine concentrations (Zouhal et al., 2013). Secondly, RT has the potential to raise basic energy metabolism in pubertal children by increasing muscle mass (Konopka and Harber, 2014) which could increase the amount of daily caloric intake and hence reduce fat mass (Nouri et al., 2013). Therefore, adding HIIT to RT could potentiate the effects of HIIT and hence trigger additive effects in obese youth (Gäbler et al., 2018). Based on these findings, it appears that combined training is an optimal training mode to reduce body mass, BMI, and body fat.

### 4.2 Aerobic Capacity and Musculoskeletal Fitness

Cardiorespiratory fitness has been shown to be a strong predictor of morbidity and mortality (Alberga et al., 2013). Regarding the effect of different training modalities on physical fitness, the present study reported a significant increase in estimated VO_2max_ and 6 MWT-distance in the HIIT, RT and CT groups from baseline to post-intervention; however, the CT group displayed significantly greater improvements than the RT and HIIT groups.

The observation that the HIIT program resulted in a significant increase in indices of aerobic capacity is in agreement with the available literature in adolescents with obesity that showed increases in cardiorespiratory fitness following HIIT programs (Racil et al., 2013; Ouerghi et al., 2014; Lau et al., 2015; Ouerghi et al., 2017). Confirming the results of the current work, a meta-analysis conducted by García-Hermoso et al. (2016) with overweight and obese children, found that HIIT had a stronger favorable effect on aerobic capacity than other forms of exercise (moderate-intensity continuous training, moderate-intensity interval training and low-intensity interval training). Several authors have described HIIT programs as improving VO_2max_ due to an increase in oxygen availability as seen from central effects (such as maximal cardiac output, total hemoglobin, and blood plasma volume) (Astorino et al., 2012) and/or as a result of peripheral adaptive responses with an enhanced ability to extract and use available oxygen due to higher muscle oxidative potential (Burgomaster et al., 2008). Nevertheless, changes in mitochondrial enzymes in muscles alone cannot justify changes in VO_2max_ since enzymatic changes are sometimes more pronounced than improvements in this aerobic capacity variable (Burgomaster et al., 2008). Alterations in aerobic capacity indices, may be linked to body composition changes, particularly in obese people (Drigny et al., 2014). However, weight loss, is not required for exercise-induced improvements in aerobic and anaerobic capacity (Mazurek et al., 2016). Regarding the CT program, our observation was that the combination of HIIT and RT resulted in a significantly greater increase in aerobic capacity than HIIT or RT alone, which is consistent with other studies in youth with obesity (Ho et al., 2012; Sigal et al., 2014).

There have been few studies that measured the effects of HIIT on anaerobic metrics in obese people (Ouerghi et al., 2017). Our study showed that a 9-week HIIT program was effective to improve anaerobic performance among collegians with obesity. These findings agree with Ouerghi et al. (2017) who demonstrated significant enhancements in anaerobic markers (vertical jump performance; 10-, 30-m sprint speed) after an 8-week HIIT program in obese adolescents. The improvement could be attributed to enhanced muscle glycogenolytic and anaerobic glycolytic enzyme activity following interval training (Ouerghi et al., 2017). An increase in cardiorespiratory fitness after RT in the present study, does not concur with Lee et al.’s (2013) study, which evaluated performance indicators in 44 obese adolescent girls who were randomly assigned to aerobic or RT. In this study, cardiorespiratory fitness improved by 17% in the aerobic-training group, while there were no changes in the RT group. Differences in methodology, study duration, adherence, sample characteristics, and supervision intensity could all have a role in this difference of results.

According to our findings, all three types of exercise training (HIIT, RT, and CT) increased upper and lower body muscular strength. These improved lower body muscular strength results with obese adolescents are in agreement with overall increases with HIIT, RT and CT found by Alberga et al. (2015) as well as with aerobic-exercise interval-training (Tjønna et al., 2009). However, the findings contradict Lee et al. (2012) who found no significant changes in chest or leg strength following aerobic-exercise training in obese male adolescents compared to a non-exercising control group.

Moreover, in the present study, leg power (assessed from the CMJ test) and handgrip strength were increased in a greater extent following RT than HIIT and CT. Our findings are consistent with those of the Alberga et al.’s (2015) study showing a greater improvement in lower body muscular strength (leg press) in the RT group. Our results are also in agreement with those of the Lee et al.’s (2013) study, reporting a 45% improvement in a muscular-strength index (the sum of 1-RM chest and leg press scores expressed per kilogram of body mass) in the RT group compared to the non-exercising controls in obese adolescent girls. Conversely, the results of the vertical jump test that Alberga et al. (2015) had used to assess leg power did not change following exercise training in any group. Whereas neural adaptations were not evaluated, it has been suggested that RT programs of 4–12 weeks emphasize neural adaptations such as increases in motor neuron activation (recruitment and rate coding) and motor unit synchronization (Behm, 1995), which could have contributed to the improvements in upper and lower body muscular strength in our study.

From a physiological point of view, CT, combining aerobic and resistance exercises, may have a greater effect on both myofibrillar and mitochondrial protein synthesis and oxidative capacity (Tang et al., 2006) compared to single bout of resistance or endurance exercise, leading to a greater gain in muscle size/strength and cardiovascular fitness. As a result, the findings of our study may help to enhance the health and quality of life of young obese collegians with exercise therapy alone, as well as provide them with a viable solution to deal with the problem of obesity. Potential health issues, such as cardiovascular or respiratory difficulties, can also be prevented indirectly without affecting diet.

### 4.3 Strengths and Limitations

In contrast to previous research that have investigated RT, endurance training (ET) and CT (Libardi et al., 2012; Alberga et al., 2015; Racil et al., 2016), to our knowledge, this is the first study that employed a design in which CT participants completed 50% of a RT and 50% of a HIIT at each session rather than a full session of each modality (i.e., double the dose). In addition, the present study is the first having investigated the effects of an equal volume of three training modalities (endurance, strength and CT) on body composition and performance-related responses for 9 weeks in sedentary healthy youth population with obesity. The most commonly used CT regimens have been continuous endurance and/or resistance training, rather than HIIT. Also, a uniqueness of the current investigation is that it explored the response of such CT modality in pediatric obesity.

Overall, several advantages could be noted in our study: 1) the controlled interventions performed with trained personal at the designated center CNMSS resulted in a considerable fat reduction in obese teens receiving 3 modes of training; 2) Before and following the intervention, we did control, the caloric intake, which is well acknowledged as a key factor in obesity development. 3) Furthermore, the low cost of the training and testing equipment will allow duplication in many contexts, including schools, sports centers, clubs and basic health units. This may provide an economic health promotion strategy.

Some limitations are however acknowledged in this study. Firstly, cycling can be a more appropriate method of exercise rather than running on a track in view of orthopedic problems in obese populations. However, the running exercise can be done without an ergometer and thus in everyday life. Secondly, we did not use a control group; this is due on the one hand because the scientific literature has clearly demonstrated the effectiveness of each training modality (HIIT, RT or CT) for improving body composition and physical performance when it is practiced separately in such a population. Finally, BMI has known limitations, such as failure to distinguish fat mass from lean mass, even WHO uses the BMI though. The ability to correctly identify obesity in a population is still debated (Frankenfield et al., 2001). In our study, body fat was also assessed using DEXA and the results indicated that the enrolled subjects all had a % body fat >30% (Table 4).

## 5 Conclusion

Based on the results of the current study, all training modalities proved to be beneficial for body mass loss, BMI and fat loss; CT was more effective to improve a range of health-related parameters, and provided greater benefits for fat loss, body mass loss and cardiorespiratory fitness than HIIT or RT modalities alone. Accordingly, concurrent training should be recommended for sedentary obese youth in National Physical Activity Guideline.

## Data Availability

The raw data supporting the conclusions of this article will be made available by the authors, without undue reservation.

## References

[B85] Abdi H. Williams L. J. (2010). Contrast Analysis. Encycl. Res. Des. 1, 243–251.

[B1] Agostinis‐Sobrinho C. A. Moreira C. Abreu S. Lopes L. Sardinha L. B. Oliveira‐Santos J. (2017). Muscular Fitness and Metabolic and Inflammatory Biomarkers in Adolescents: Results from LabMed Physical Activity Study. Scand. J. Med. Sci. Sports 27, 1873–1880. 10.1111/sms.12805 27882600

[B2] Alberga A. S. Frappier A. Sigal R. J. Prud'homme D. Kenny G. P. (2013). A Review of Randomized Controlled Trials of Aerobic Exercise Training on Fitness and Cardiometabolic Risk Factors in Obese Adolescents. Phys. Sportsmed. 41, 44–57. 10.3810/psm.2013.05.2014 23703517

[B3] Alberga A. S. Prud’homme D. Sigal R. J. Goldfield G. S. Hadjiyannakis S. Phillips P. (2015). Effects of Aerobic Training, Resistance Training, or Both on Cardiorespiratory and Musculoskeletal Fitness in Adolescents with Obesity: The HEARTY Trial. Appl. Physiol. Nutr. Metab. 41, 255–265. 10.1139/apnm-2015-0413 26881317

[B4] Alkahtani S. A. King N. A. Hills A. P. Byrne N. M. (2013). Effect of Interval Training Intensity on Fat Oxidation, Blood Lactate and the Rate of Perceived Exertion in Obese Men. Springerplus 2, 532. Available at: http://www.springerplus.com/content/2/1/532 . 10.1186/2193-1801-2-532 24255835 PMC3824717

[B5] Ammar A. Karim O. A. Chtourou H. Parish A. Hoekelmann A. (2015). Prevalence of Overweight and Obesity and Possible Effect of Intervention Program: Tunisian Children as Model. Sport Sci. Health 11, 129–136. 10.1007/s11332-015-0224-2

[B6] Astorino T. A. Allen R. P. Roberson D. W. Jurancich M. (2012). Effect of High-Intensity Interval Training on Cardiovascular Function, V̇o2max, and Muscular Force. J. Strength Cond. Res. 26, 138–145. 10.1519/JSC.0b013e318218dd77 22201691

[B7] ATS (2002). American Thoracic Society ATS Statement: Guidelines for the Six-Minute Walk Test. Am. J. Respir. Crit. CARE Med. 166, 111–117. 10.1164/rccm.166/1/111 12091180

[B8] Baquet G. Berthoin S. Dupont G. Blondel N. Fabre C. Van Praagh E. (2002). Effects of High Intensity Intermittent Training on Peak V ˙ O 2 in Prepubertal Children. Int. J. Sports Med. 23, 439–444. 10.1055/s-2002-33742 12215964

[B9] Barry V. W. Baruth M. Beets M. W. Durstine J. L. Liu J. Blair S. N. (2014). Fitness vs. Fatness on All-Cause Mortality: a Meta-Analysis. Prog. Cardiovasc. Dis. 56, 382–390. 10.1016/J.PCAD.2013.09.002 24438729

[B10] Behm D. G. (1995). Neuromuscular Implications and Applications of Resistance Training. J. Strength Cond. Res. 9, 264–274. 10.1519/00124278-199511000-00014

[B11] Bull F. C. Al-Ansari S. S. Biddle S. Borodulin K. Buman M. P. Cardon G. (2020). World Health Organization 2020 Guidelines on Physical Activity and Sedentary Behaviour. Br. J. Sports Med. 54, 1451–1462. 10.1136/bjsports-2020-102955 33239350 PMC7719906

[B12] Burgomaster K. A. Howarth K. R. Phillips S. M. Rakobowchuk M. Macdonald M. J. Mcgee S. L. (2008). Similar Metabolic Adaptations during Exercise after Low Volume Sprint Interval and Traditional Endurance Training in Humans. J. Physiol. 586, 151–160. 10.1113/jphysiol.2007.142109 17991697 PMC2375551

[B13] Chaouachi A. Hammami R. Kaabi S. Chamari K. Drinkwater E. J. Behm D. G. (2014). Olympic Weightlifting and Plyometric Training with Children Provides Similar or Greater Performance Improvements Than Traditional Resistance Training. J. Strength Cond. Res. 28, 1483–1496. 10.1519/JSC.0000000000000305 24172724

[B14] Cherif L. Ayadi H. Khmakhem K. Kacem I. H. Kammoun S. Moalla Y. (2018). Problematic Video Game Use Among Teenagers in Sfax, Tunisia. J. Health Edu Res. Dev. 06. 10.4172/2380-5439.1000268

[B15] Coffey V. G. Hawley J. A. (2017). Concurrent Exercise Training: Do Opposites Distract? J. Physiol. 595, 2883–2896. 10.1113/JP272270 27506998 PMC5407958

[B16] Cohen J. (1988). Statistical Power Analysis for the Behavioral Sciences (2nd Edition).. Hillsdale, NJ: Erlbaum, 817.

[B17] De Onis M. Onyango A. W. Borghi E. Siyam A. Nishida C. Siekmann J. (2007). Development of a WHO Growth Reference for School-Aged Children and Adolescents ‬. Bull. World Health Organ 85, 660–667. 10.2471/BLT.07.043497 18026621 PMC2636412

[B18] Donges C. Timmons J. F. Minnock D. Hone M. Cogan K. E. Murphy J. C. (2013). Comparative Effects of Single-Mode vs. Duration-Matched Concurrent Exercise Training on Body Composition, Low-Grade Inflammation, and Glucose Regulation in Sedentary, Overweight Middle-Aged Men. Scand. J. Med. Sci. Sports 28, 2272–2283. 10.1111/sms.13254

[B19] Donnelly J. E. Honas J. J. Smith B. K. Mayo M. S. Gibson C. A. Sullivan D. K. (2013). Aerobic Exercise Alone Results in Clinically Significant Weight Loss for Men and Women: Midwest Exercise Trial 2. Obesity 21, E219–E228. 10.1002/oby.20145 23592678 PMC3630467

[B20] Drigny J. Gremeaux V. Dupuy O. Gayda M. Bherer L. Juneau M. (2014). Effect of Interval Training on Cognitive Functioning and Cerebral Oxygenation in Obese Patients: A Pilot Study. J. Rehabil. Med. 46, 1050–1054. 10.2340/16501977-1905 25297458

[B21] Elloumi M. Makni E. Ounis O. B. Moalla W. Zbidi A. Zaoueli M. (2011). Six-minute Walking Test and the Assessment of Cardiorespiratory Responses during Weight-Loss Programmes in Obese Children. Physiother. Res. Int. 16, 32–42. 10.1002/pri.470 21110411

[B22] Farrag N. S. Cheskin L. J. Farag M. K. (2017). A Systematic Review of Childhood Obesity in the Middle East and North Africa (MENA) Region: Prevalence and Risk Factors Meta-Analysis. Adv. Pediatr. Res. 4, 8. 10.12715/apr.2017.4.8 29354689 PMC5773115

[B23] Faul F. Erdfelder E. Lang A.-G. Buchner A. (2007). G*Power 3: A Flexible Statistical Power Analysis Program for the Social, Behavioral, and Biomedical Sciences. Behav. Res. Methods 39, 175–191. 10.3758/BF03193146 17695343

[B24] Fillon A. Miguet M. O’Malley G. Mathieu M.-E. Masurier J. Julian V. (2020). Is the SPARTACUS 15-15 Test an Accurate Proxy for the Assessment and Tracking of Maximal Aerobic Capacities in Adolescents with Obesity? J. Phys. Ther. Sci. 32, 281–287. 10.1589/jpts.32.281 32273651 PMC7113421

[B25] Foster C. Florhaug J. A. Franklin J. Gottschall L. Hrovatin L. A. Parker S. (2001). A New Approach to Monitoring Exercise Training. J. Strength Cond. Res. 15, 109–115. 10.1519/00124278-200102000-00019 11708692

[B26] Frankenfield D. C. Rowe W. A. Cooney R. N. Smith J. S. Becker D. (2001). Limits of Body Mass Index to Detect Obesity and Predict Body Composition. Nutrition 17, 26–30. 10.1016/S0899-9007(00)00471-8 11165884

[B27] Gäbler M. Prieske O. Hortobágyi T. Granacher U. (2018). The Effects of Concurrent Strength and Endurance Training on Physical Fitness and Athletic Performance in Youth: A Systematic Review and Meta-Analysis. Front. Physiol. 9, 1057. 10.3389/fphys.2018.01057 30131714 PMC6090054

[B28] Gaha R. Ghannem H. Harrabi I. Ben Abdelazi A. Lazreg F. Hadj Fredj A. (2002). Étude de la surcharge pondérale et de l'obésité dans une population d'enfants et d'adolescents scolarisés en milieu urbain à Sousse en Tunisie. Arch. Pédiatrie 9, 566–571. 10.1016/S0929-693X(01)00922-8 12108309

[B29] Garber C. E. Blissmer B. Deschenes M. R. Franklin B. A. Lamonte M. J. Lee I.-M. (2011). Quantity and Quality of Exercise for Developing and Maintaining Cardiorespiratory, Musculoskeletal, and Neuromotor Fitness in Apparently Healthy Adults: Guidance for Prescribing Exercise. Med. Sci. Sports Exerc. 43, 1334–1359. 10.1249/MSS.0b013e318213fefb 21694556

[B30] García-Hermoso A. Cerrillo-Urbina A. J. Herrera-Valenzuela T. Cristi-Montero C. Saavedra J. M. Martínez-Vizcaíno V. (2016). Is High-Intensity Interval Training More Effective on Improving Cardiometabolic Risk and Aerobic Capacity Than Other Forms of Exercise in Overweight and Obese Youth? A Meta-Analysis. Obes. Rev. 17, 531–540. 10.1111/obr.12395 26948135

[B31] García-Hermoso A. Ramírez-Vélez R. Ramírez-Campillo R. Peterson M. D. Martínez-Vizcaíno V. (2018). Concurrent Aerobic Plus Resistance Exercise versus Aerobic Exercise Alone to Improve Health Outcomes in Paediatric Obesity: A Systematic Review and Meta-Analysis. Br. J. Sports Med. 52, 161–166. 10.1136/bjsports-2016-096605 27986760

[B32] Ghouili H. Ben Khalifa W. Ouerghi N. Zouaoui M. Dridi A. Gmada N. (2018). Body Mass Index Reference Curves for Tunisian Children. Arch. Pédiatrie 25, 459–463. 10.1016/j.arcped.2018.09.005 30361086

[B33] Groslambert A. Hintzy F. Hoffman M. D. Duguél B. Rouillon J. D. (2001). Validation of a Rating Scale of Perceived Exertion in Young Children. Int. J. Sports Med. 22, 116–119. 10.1055/S-2001-11340 11281613

[B34] Guedes D. P. Lopes C. C. Guedes J. E. R. P. (2005). Reprodutibilidade e validade Do Questionário Internacional de Atividade Física em adolescentes. Rev. Bras. Med. Esporte 11, 151–158. 10.1590/S1517-86922005000200011

[B35] Hammami R. Chaouachi A. Makhlouf I. Granacher U. Behm D. G. (2016). Associations between Balance and Muscle Strength, Power Performance in Male Youth Athletes of Different Maturity Status. Pediatr. Exerc Sci. 28, 521–534. 10.1123/pes.2015-0231 27046937

[B36] Hardy L. L. Mihrshahi S. Bellew W. Bauman A. Ding D. (2017). Children's Adherence to Health Behavior Recommendations Associated with Reducing Risk of Non-communicable Disease. Prev. Med. Rep. 8, 279–285. 10.1016/j.pmedr.2017.10.006 29255663 PMC5723372

[B37] Ho S. S. Dhaliwal S. S. Hills A. P. Pal S. (2012). The Effect of 12 Weeks of Aerobic, Resistance or Combination Exercise Training on Cardiovascular Risk Factors in the Overweight and Obese in a Randomized Trial. BMC Public Health 12, 704. 10.1186/1471-2458-12-704 23006411 PMC3487794

[B38] Hopkins W. G. Marshall S. W. Batterham A. M. Hanin J. (2009). Health Effects of Overweight and Obesity in 195 Countries over 25 Years. N. Engl. J. Med. 377, 13–27. 10.1056/nejmoa1614362 PMC5477817 28604169

[B39] Hruby A. Hu F. B. (2015). The Epidemiology of Obesity: A Big Picture. Pharm. Econ. 33, 673–689. 10.1007/s40273-014-0243-x PMC4859313 25471927

[B40] Hsu C.-Y. Chen L.-S. Chang I.-J. Fang W.-C. Huang S.-W. Lin R.-H. (2021). Can Anthropometry and Body Composition Explain Physical Fitness Levels in School-Aged Children? Children 8, 460. 10.3390/children8060460 34072785 PMC8229107

[B41] Jamka M. Mądry E. Wasiewicz-Gajdzis M. Bajerska J. Kokot M. Kaczmarek N. (2021). The Effect of Endurance and Endurance-Strength Training on Body Composition and Cardiometabolic Markers in Abdominally Obese Women: a Randomised Trial. Available at: https://www.nature.com/articles/s41598-021-90526-7 (Accessed July 8, 2021). 10.1038/s41598-021-90526-7 PMC8196030 34117276

[B42] Knöpfli B. H. Radtke T. Lehmann M. Schätzle B. Eisenblätter J. Gachnang A. (2008). Effects of a Multidisciplinary Inpatient Intervention on Body Composition, Aerobic Fitness, and Quality of Life in Severely Obese Girls and Boys. J. Adolesc. Health 42, 119–127. 10.1016/j.jadohealth.2007.08.015 18207089

[B43] Konopka A. R. Harber M. P. (2014). Skeletal Muscle Hypertrophy after Aerobic Exercise Training. Exerc. Sport Sci. Rev. 42, 53–61. 10.1249/JES.0000000000000007 24508740 PMC4523889

[B44] Lau P. W. C. Wong D. P. Ngo J. K. Liang Y. Kim C. G. Kim H. S. (2015). Effects of High-Intensity Intermittent Running Exercise in Overweight Children. Eur. J. Sport Sci. 15, 182–190. 10.1080/17461391.2014.933880 25012183

[B45] Lee S. Bacha F. Hannon T. Kuk J. L. Boesch C. Arslanian S. (2012). Effects of Aerobic versus Resistance Exercise without Caloric Restriction on Abdominal Fat, Intrahepatic Lipid, and Insulin Sensitivity in Obese Adolescent Boys. Diabetes 61, 2787–2795. 10.2337/db12-0214 22751691 PMC3478522

[B46] Lee S. Deldin A. R. White D. Kim Y. Libman I. Rivera-Vega M. (2013). Aerobic Exercise but Not Resistance Exercise Reduces Intrahepatic Lipid Content and Visceral Fat and Improves Insulin Sensitivity in Obese Adolescent Girls: A Randomized Controlled Trial. Am. J. Physiol.-Endocrinol. Metabol. 305, E1222–E1229. 10.1152/ajpendo.00285.2013 PMC3840217 24045865

[B47] Liao C.-D. Tsauo J.-Y. Lin L.-F. Huang S.-W. Ku J.-W. Chou L.-C. (2017). Effects of Elastic Resistance Exercise on Body Composition and Physical Capacity in Older Women with Sarcopenic Obesity: a CONSORT-Compliant Prospective Randomized Controlled Trial. Medicine 96, e7115. 10.1097/MD.0000000000007115 28591061 PMC5466239

[B90] Libardi C. A. De Souza G. V. Cavaglieri C. R. Madruga V. A. Chacon-Mikahil M. P. T. (2012). Effect of Resistance, Endurance, and Concurrent Training on TNF-α, IL-6, and CRP. Medicine and Science in Sports and Exercise 44, 5056. 10.1249/MSS.0b013e318229d2e9 21697747

[B48] Lopes J. S. S. Machado A. F. Micheletti J. K. de Almeida A. C. Cavina A. P. Pastre C. M. (2019). Effects of Training with Elastic Resistance versus Conventional Resistance on Muscular Strength: A Systematic Review and Meta-Analysis. SAGE Open Med. 7, 205031211983111. 10.1177/2050312119831116 PMC6383082 30815258

[B49] Maatoug J. M. Harrabi I. Delpierre C. Gaha R. Ghannem H. (2013). Predictors of Food and Physical Activity Patterns Among Schoolchildren in the Region of Sousse, Tunisia. Obes. Res. Clin. Pract. 7, e407–e413. 10.1016/j.orcp.2012.05.006 24304483

[B50] Magnani Branco B. H. Carvalho I. Z. Garcia de Oliveira H. Fanhani A. P. Machado Dos Santos M. C. Pestillo de Oliveira L. (2020). Effects of 2 Types of Resistance Training Models on Obese Adolescents' Body Composition, Cardiometabolic Risk, and Physical Fitness. J. strength Cond. Res. 34, 2672–2682. 10.1519/JSC.0000000000002877 30557175

[B51] Makhlouf I. Chaouachi A. Chaouachi M. Ben Othman A. Granacher D. G. Behm D. G. (2018). Combination of Agility and Plyometric Training Provides Similar Training Benefits as Combined Balance and Plyometric Training in Young Soccer Players. Front. Physiol. 9, 1–17. 10.3389/fphys.2018.01611 30483158 PMC6243212

[B52] Mazurek K. Zmijewski P. Krawczyk K. Czajkowska A. Kęska A. Kapuscinski P. (2016). High Intensity Interval and Moderate Continuous Cycle Training in a Physical Education Programme Improves Health-Related Fitness in Young Females. Biol. Sport 33, 139–144. 10.5604/20831862.1198626 27274106 PMC4885624

[B53] McCarthy H. D. Cole T. J. Fry T. Jebb S. A. Prentice A. M. (2006). Body Fat Reference Curves for Children. Int. J. Obes. 30, 598–602. 10.1038/sj.ijo.0803232 16570089

[B54] Monteiro P. A. Chen K. Y. Lira F. S. Saraiva B. T. C. Antunes B. M. M. Campos E. Z. (2015). Concurrent and Aerobic Exercise Training Promote Similar Benefits in Body Composition and Metabolic Profiles in Obese Adolescents. Lipids Health Dis. 14, 153. 10.1186/s12944-015-0152-9 26611872 PMC4660803

[B55] Nakhostin-roohi B. Branch A. (2018). The Effect of Concurrent Training Program on Body Composition Indices in Overweight and Obese Female Students Babak. J. Exerc. Physiol. Health 1, 6–12.

[B56] Norton K. I. (2018). “Standards for Anthropometry Assessment,” in Kinanthropometry and Exercise Physiology (Routledge), 68–137. 10.4324/9781315385662-4

[B57] Nouri Y. Rahmani Nia F. Mirzaie B. Arazi H. (2013). The Effect of Resistance and Endurance Training on Resting Metabolic Rate and Body Composition in Sedentary Males. J. Zanjan Univ. Med. Sci. Health Serv. 21, 51–63. Available at: http://zums.ac.ir/journal/article-1-2403-en.html (Accessed September 13, 2021). 10.12816/0000210

[B58] O'Donoghue G. Blake C. Cunningham C. Lennon O. Perrotta C. (2021). What Exercise Prescription Is Optimal to Improve Body Composition and Cardiorespiratory Fitness in Adults Living with Obesity? A Network Meta‐analysis. Obes. Rev. 22, 1–19. 10.1111/obr.13137 PMC7900983 32896055

[B59] Ortega F. B. Ruiz J. R. Castillo M. J. Sjöström M. (2008). Physical Fitness in Childhood and Adolescence: A Powerful Marker of Health. Int. J. Obes. 32, 1–11. 10.1038/sj.ijo.0803774 18043605

[B60] Ortega F. B. Lavie C. J. Blair S. N. (2016). Obesity and Cardiovascular Disease. Circ. Res. 118, 1752–1770. 10.1161/CIRCRESAHA.115.306883 27230640

[B61] Ortega F. B. Ruiz J. R. Labayen I. Lavie C. J. Blair S. N. (2018). The Fat but Fit Paradox: what We Know and Don't Know about it. Br. J. Sports Med. 52, 151–153. 10.1136/bjsports-2016-097400 28583992

[B62] Ouerghi N. Feki M. Kaabachi N. Khammassi M. Boukorraa S. Bouassida A. (2014). Effects of a High-Intensity Intermittent Training Program on Aerobic Capacity and Lipid Profile in Trained Subjects. Oajsm 5, 243. 10.2147/oajsm.s68701 25378960 PMC4207574

[B63] Ouerghi N. Fradj M. K. B. Bezrati I. Khammassi M. Feki M. Kaabachi N. (2017). Effects of High-Intensity Interval Training on Body Composition, Aerobic and Anaerobic Performance and Plasma Lipids in Overweight/obese and Normal-Weight Young Men. Biol. Sport 34, 385–392. 10.5114/biolsport.2017.69827 29472742 PMC5819474

[B64] Pazzianotto-Forti E. M. Moreno M. A. Plater E. Baruki S. B. S. Rasera-Junior I. Reid W. D. (2020). Impact of Physical Training Programs on Physical Fitness in People with Class II and III Obesity: A Systematic Review and Meta-Analysis. Phys. Ther. 100, 963–978. 10.1093/PTJ/PZAA045 32211862

[B65] Racil G. Ben Ounis O. Hammouda O. Kallel A. Zouhal H. Chamari K. (2013). Effects of High vs. Moderate Exercise Intensity during Interval Training on Lipids and Adiponectin Levels in Obese Young Females. Eur. J. Appl. Physiol. 113, 2531–2540. 10.1007/s00421-013-2689-5 23824463

[B66] Racil G. Lemaire C. Dubart A. E. Garcin M. Coquart J. B. (2015). Intermittent Exercise Is Beneficial to Obese Women Independently of Obesity Class. Jacobs J. Physiother. Exerc.. 1 (1), 003.

[B67] Racil G. Zouhal H. Elmontassar W. Abderrahmane A. B. De Sousa M. V. Chamari K. (2016). Plyometric Exercise Combined with High-Intensity Interval Training Improves Metabolic Abnormalities in Young Obese Females More So Than Interval Training Alone. Appl. Physiol. Nutr. Metab. 41, 103–109. 10.1139/apnm-2015-0384 26701117

[B68] Rey O. Rossi D. Nicol C. Mercier C.-S. Vallier J.-M. Maïano C. (2013). Évaluation indirecte de la capacité aérobie d’adolescents obèses: Intérêt d’un test de course à pied intermittent court, progressif et maximal. Sci. Sports 28, e133–e139. 10.1016/j.scispo.2013.02.006

[B69] Rey O. Maïano C. Nicol C. Mercier C. S. Vallier J. M. (2016). Psycho-physiological Responses of Obese Adolescents to an Intermittent Run Test Compared with a 20-m Shuttle Run. J. Sports Sci. Med. 15, 451–459. Available at: https://www.ncbi.nlm.nih.gov/pubmed/27803623 (Accessed November 12, 2019). 27803623 PMC4974857

[B70] Saeidi A. Haghighi M. M. Kolahdouzi S. Daraei A. Abderrahmane A. B. Essop M. F. (2020). The Effects of Physical Activity on Adipokines in Individuals with Overweight/obesity across the Lifespan: A Narrative Review. Obes. Rev. 22, e13090. 10.1111/obr.13090 32662238

[B71] Sasaki H. Morishima T. Hasegawa Y. Mori A. Ijichi T. Kurihara T. (2014). 4 Weeks of High-Intensity Interval Training Does Not Alter the Exercise-Induced Growth Hormone Response in Sedentary Men. SpringerPlus 3, 336. 10.1186/2193-1801-3-336 25806146 PMC4363223

[B72] Schranz N. Tomkinson G. Olds T. Olds O. (2013). What Is the Effect of Resistance Training on the Strength, Body Composition and Psychosocial Status of Overweight and Obese Children and Adolescents? A Systematic Review and Meta-Analysis. Sports Med. 43, 893–907. 10.1007/s40279-013-0062-9 23729196

[B73] Schroeder E. C. Franke W. D. Sharp R. L. Leechul D.-c. (2019). Comparative Effectiveness of Aerobic, Resistance, and Combined Training on Cardiovascular Disease Risk Factors: A Randomized Controlled Trial. PLoS ONE 14, e0210292. 10.1371/journal.pone.0210292 30615666 PMC6322789

[B74] Shultz S. P. Anner J. Hills A. P. (2009). Paediatric Obesity, Physical Activity and the Musculoskeletal System. Obes. Rev. official J. Int. Assoc. Study Obes. 10, 576–582. 10.1111/J.1467-789X.2009.00587.X 19460114

[B75] Sigal R. J. Alberga A. S. Goldfield G. S. Prud’homme D. Hadjiyannakis S. Gougeon R. (2014). Effects of Aerobic Training, Resistance Training, or Both on Percentage Body Fat and Cardiometabolic Risk Markers in Obese Adolescents: The Healthy Eating Aerobic and Resistance Training in Youth Randomized Clinical Trial. JAMA Pediatr. 168, 1006–1014. 10.1001/jamapediatrics.2014.1392 25243536

[B76] Silva J. R. (2019). Concurrent Aerobic and Strength Training for Performance in Soccer, 397, 416. 10.1007/978-3-319-75547-2_27

[B77] Tang J. E. Hartman J. W. Phillips S. M. (2006). Increased Muscle Oxidative Potential Following Resistance Training Induced Fibre Hypertrophy in Young Men. Appl. Physiol. Nutr. Metab. 31, 495–501. 10.1139/H06-026 17111003

[B78] Thivel D. O'''''Malley G. Blourdier D. Tremeaux M. Zanchet C. Pereira B. (2017). Reproducibility of the Intermittent Spartacus Run Test in Obese Adolescents. J. Sports Med. Phys. Fit. 57, 1083–1088. 10.23736/S0022-4707.16.06534-8 27387496

[B79] Thivel D. (2011). Acute Exercise and Subsequent Energy Balance : Interest in Obese Youths. Available at: https://hal.uca.fr/tel-01342620v2 (Accessed December 20, 2021).

[B80] Tjønna A. E. Stølen T. O. Bye A. Volden M. Slørdahl S. A. Ødegård R. (2009). Aerobic Interval Training Reduces Cardiovascular Risk Factors More Than a Multitreatment Approach in Overweight Adolescents. Clin. Sci. 116, 317–326. 10.1042/CS20080249 18673303

[B81] Türk Y. Theel W. Kasteleyn M. J. Franssen F. M. E. Hiemstra P. S. Rudolphus A. (2017). High Intensity Training in Obesity: a Meta-Analysis. Obes. Sci. Pract. 3, 258–271. 10.1002/osp4.109 29071102 PMC5598019

[B91] Van Den Tillaar R. Mário C. (2013). Reliability of Seated and Standing Throwing Velocity Using Differently Weighted Medicine Balls. Journal of Strength and Conditioning Research 27, 12341238. 10.1519/JSC.0b013e3182654a09 22744301

[B82] Vanhelst J. Fardy P. S. Salleron J. Béghin L. (2013). The Six-Minute Walk Test in Obese Youth: Reproducibility, Validity, and Prediction Equation to Assess Aerobic Power. Disabil. Rehabil. 35, 479–482. 10.3109/09638288.2012.699581 22779759

[B83] Vechin F. C. Conceição M. S. Telles G. D. Libardi C. A. Ugrinowitsch C. (2021). Interference Phenomenon with Concurrent Strength and High-Intensity Interval Training-Based Aerobic Training: An Updated Model. Sports Med. 51, 599–605. 10.1007/s40279-020-01421-6 33405189

[B84] Wearing S. C. Hennig E. M. Byrne N. M. Steele J. R. Hills A. P. (2006). The Impact of Childhood Obesity on Musculoskeletal Form. Obes. Rev. 7, 209–218. 10.1111/j.1467-789X.2006.00216.x 16629876

[B86] Wu N. Chen Y. Yang J. Li F. (2017). Childhood Obesity and Academic Performance: The Role of Working Memory. Front. Psychol. 8, 611. 10.3389/fpsyg.2017.00611 28469593 PMC5395561

[B87] Zhang H. Tong T. K. Qiu W. Zhang X. Zhou S. Liu Y. (2017). Comparable Effects of High-Intensity Interval Training and Prolonged Continuous Exercise Training on Abdominal Visceral Fat Reduction in Obese Young Women. J. Diabetes Res. 2017, 1–9. 10.1155/2017/5071740 PMC5237463 28116314

[B88] Zouhal H. Lemoine-Morel S. Mathieu M.-E. Casazza G. A. Jabbour G. (2013). Catecholamines and Obesity: Effects of Exercise and Training. Sports Med. 43, 591–600. 10.1007/s40279-013-0039-8 23613311

[B89] Zouhal H. Ben Abderrahman A. Khodamoradi A. Saeidi A. Jayavel A. Hackney A. C. (2020). Effects of Physical Training on Anthropometrics, Physical and Physiological Capacities in Individuals with Obesity: A Systematic Review. Obes. Rev. 21, e13039. 10.1111/obr.13039 32383553

